# Strengthening Jordan’s Laboratory Capacity for Communicable Diseases: A Comprehensive Multi-Method Mapping Toward Harmonized National Laboratories and Evidence-Informed Public Health Planning

**DOI:** 10.3390/ijerph22091459

**Published:** 2025-09-20

**Authors:** Dalia Kashef Zayed, Ruba A. Al-Smadi, Mohammad Almaayteh, Thekryat Al-Hjouj, Ola Hamdan, Ammar Abu Ghalyoun, Omar Alsaleh, Tariq Abu Touk, Saddam Nawaf Almaseidin, Thaira Madi, Samar Khaled Hassan, Muna Horabi, Adel Belbiesi, Tareq L. Mukattash, Ala’a B. Al-Tammemi

**Affiliations:** 1 Jordan Center for Disease Control, Amman 11183, Jordan; d.zayed@jcdc.gov.jo (D.K.Z.); r.alsmadi@jcdc.gov.jo (R.A.A.-S.); mohd_maaita@outlook.com (M.A.); z.khaled@jcdc.gov.jo (T.A.-H.); o.hamdan@jcdc.gov.jo (O.H.); a.abughalyoun@jcdc.gov.jo (A.A.G.); o.kamal@jcdc.gov.jo (O.A.); t.hisham@jcdc.gov.jo (T.A.T.); s.nawaf@jcdc.gov.jo (S.N.A.); m.horabi@jcdc.gov.jo (M.H.); a.belbaisi@jcdc.gov.jo (A.B.); t.mukattash@jcdc.gov.jo (T.L.M.); 2 Princess Iman Center for Research and Laboratory, Royal Medical Services, Amman 11855, Jordan; 3 Department of Accreditation, Health Care Accreditation Council, Amman 11181, Jordan; talmadi@hcac.com.jo (T.M.); shassan@hcac.com.jo (S.K.H.); 4 Department of Clinical Pharmacy, Faculty of Pharmacy, Jordan University of Science and Technology, Irbid 22110, Jordan

**Keywords:** Jordan, laboratory systems, communicable diseases, One Health, quality management, health security, mapping, diagnostic capacity, public health

## Abstract

Infectious diseases remain a global threat, with low- and middle-income countries disproportionately affected due to socio-economic and demographic vulnerabilities. Robust laboratory systems are critical for early detection, outbreak containment, and guiding effective interventions. This study aimed to map and evaluate Jordan’s laboratory diagnostic network for communicable diseases, identify gaps, and recommend strategies to strengthen capacity, harmonization, and alignment with international standards. A multi-method approach was employed in 2023 through collaboration between the Jordan Center for Disease Control and the Health Care Accreditation Council. Data were collected via (i) a desktop review of 226 national and international documents; (ii) 20 key informant interviews with stakeholders from the public, private, military, veterinary, and academic sectors; and (iii) 23 field visits across 27 laboratories in four Jordanian governorates. Data were analyzed thematically and synthesized using the LABNET framework, which outlined ten core laboratory capacities. Findings were validated through a multi-sectoral national workshop with 90 participants. The mapping revealed the absence of a unified national laboratory strategic plan, with governance dispersed across multiple authorities and limited inter-sectoral coordination. Standard operating protocols (SOPs) existed for high-priority diseases such as T.B, HIV, influenza, and COVID-19 but were lacking or outdated for other notifiable diseases, particularly zoonoses. Quality management was inconsistent, with limited participation in external quality assurance programs and minimal accreditation uptake. Biosafety and biosecurity frameworks were fragmented and insufficiently enforced, while workforce shortages, high turnover, and limited specialized training constrained laboratory performance. Despite these challenges, Jordan demonstrated strengths including skilled laboratory staff, established reference centers, and international collaborations, which provide a platform for improvement. Jordan’s laboratory network has foundational strengths but faces systemic challenges in policy coherence, standardization, quality assurance, and workforce capacity. Addressing these gaps requires the development of a national laboratory strategic plan, strengthened legal and regulatory frameworks, enhanced quality management and accreditation, and integrated One Health coordination across human, animal, and environmental health sectors. These measures will improve diagnostic reliability, preparedness, and alignment with the global health security agenda.

## 1. Introduction

In recent decades, the world has experienced numerous outbreaks and epidemics of infectious diseases with the potential for global spread. Although high-income and low- and middle-income (LMICs) countries are straggling with the burden of infectious diseases, LMICs are particularly exposed and vulnerable due to various demographic and socio-economic factors [1,2,3].

Laboratories play a vital role in the detection, identification, and monitoring of emerging infectious diseases. The COVID-19 pandemic clearly demonstrated this, as countries with robust laboratory systems were able to implement rapid widespread testing, an essential step for tracking infections and informing effective public health interventions [4].

When infectious diseases go undiagnosed in their early stages, the delay in identifying and responding to them can seriously hinder containment efforts, allowing the disease to spread more widely and linger longer. In today’s interconnected healthcare landscape, early detection is not just a clinical tool, it is a lifesaving strategy. It can lead to better patient outcomes, lower healthcare costs, and ultimately, a higher quality of life for individuals and societies alike.

Public health preparedness depends on the strength and readiness of national laboratory systems [5,6,7,8]. These laboratories are essential for swiftly diagnosing infections to guide timely treatment, detecting and containing outbreaks, and characterizing infectious agents to support the development of effective vaccines and control strategies. Additionally, they play a critical role in monitoring the effectiveness of prevention efforts and tracking the containment of antimicrobial resistance (AMR), noting that AMR represents one of the most urgent global health threats, contributing to millions of deaths annually worldwide. In Jordan, national assessments have shown progress in developing policies but persistent challenges in laboratory capacity for antimicrobial susceptibility testing and surveillance. Effective AMR control depends on standardized laboratory standard operating procedures (SOPs), robust quality assurance, and integration into national and global surveillance platforms [9]. Strengthening AMR diagnostic capacity within the laboratory system is therefore an essential component of communicable disease preparedness. Taking into consideration that about 70–75% of medical diagnoses are obtained via clinical laboratory reports, making laboratory service quality directly impact healthcare quality [10]. Additionally, quality control and assurance measures are often inadequately implemented or insufficiently monitored, which can undermine the reliability and accuracy of laboratory results. These challenges underscore the urgent need for a comprehensive review of existing laboratory-related policies and procedures to ensure consistency, operational efficiency, and alignment with national, regional, and international standards.

Laboratory SOPs are fundamental to ensuring robust biosafety and biosecurity measured [11]. In Jordan, a middle-income country in the Eastern Mediterranean Region (EMR), basic biosafety protocols are in place; nonetheless, the current national guidelines would benefit from further harmonization and updating to align with internationally recognized standards, such as the World Health Organization (WHO) Laboratory Biosafety Manual and the International Air Transport Association (IATA) regulations for the transport of infectious substances. Advancing biosafety governance in this way will contribute to safer, more reliable, and internationally compliant laboratory practices, thereby strengthening both national and regional health security [9,12].

Despite the critical role laboratories play in detecting communicable diseases, the sector faces several significant challenges. One of the key issues is the fragmentation and lack of coherence across existing frameworks, such as legislation, policies, protocols, and SOPs. SOPs, which are detailed, step-by-step guidelines for performing procedures, are designed to promote consistency, accuracy, and high-quality data. However, inconsistencies in these documents can compromise laboratory performance and the reliability of results [13]. SOPs are the main component of a laboratory quality assurance framework and are embedded in the Quality Management System (QMS). Zoonotic diseases such as brucellosis, rabies, and avian influenza continue to pose recurrent threats in Jordan, straddling the human–animal interface and requiring multisectoral laboratory coordination. Previous mapping has demonstrated critical gaps in zoonotic laboratory capacity and reporting systems. Addressing these gaps is vital for early detection and control [12,14].

Jordan has a rapidly evolving laboratory sector that is distributed across various administrative entities. This sector encompasses a wide range of services, including clinical laboratories within hospitals and independent facilities, public health laboratories dedicated to disease surveillance and outbreak response, as well as laboratories specializing in water quality, animal health, food safety, and environmental testing. The diversity and decentralization of these laboratories highlight the importance of conducting comprehensive situational analyses to assess existing policies, protocols, quality systems, and operational practices. Such analyses are crucial for identifying gaps, promoting harmonization, and strengthening the overall performance and integration of the laboratory network in Jordan [15]. This will support the establishment of a clear and cohesive framework for informed decision-making, quality assurance, and risk management, while also enabling the assessment of alignment with international best practices and standards, including those set by the WHO and the International Organization for Standardization (ISO).

In Jordan, several laboratories have developed well-documented SOPs for the identification and characterization of pathogens, many of which are aligned with international standards and references, including those from the U.S. Centers for Disease Control and Prevention (CDC), the United Kingdom’s National Health Service (NHS), and the U.S. Food and Drug Administration (FDA). Despite progress in selected laboratories, the absence of standardized SOPs, fragmented policies, and uneven accreditation underscore the need for a comprehensive national mapping and review of laboratory systems in Jordan [13].

Given the interconnectedness of human, animal, and environmental health, the One Health approach provides a unifying framework for strengthening laboratory systems. Integrating One Health principles into national laboratory policies is particularly important for Jordan, where zoonotic diseases are prevalent, AMR is increasing, and environmental risks intersect with public health. To address this gap, the Jordan Center for Disease Control (JCDC) and the Health Care Accreditation Council (HCAC) undertook a comprehensive assessment of the national laboratory diagnostic network for communicable diseases. Using the LABNET core-capacity framework adapted for One Health, we assessed Jordan’s national laboratory network for communicable diseases to: (i) quantify gaps in policy, governance, quality systems, biosafety, information systems, and workforce; (ii) identify root causes affecting performance; and (iii) generate a prioritized, phased reform agenda aligned with International Health Regulations and Global Health Security Agenda.

## 2. Methods

### 2.1. Study Design

This paper presents an in-depth evaluation of Jordan’s national laboratory diagnostic landscape for communicable diseases which was conducted in 2023. It constitutes a component of a wider national mapping initiative and was carried out through a multi-method design implemented across sequential phases. These phases included: (1) team formation, (2) stakeholder mapping, (3) technical committee formation, (4) tool development and data collection, (5) data analysis and evaluation, and (6) validation workshop. This design was chosen to ensure triangulation of data sources and to capture both documentary evidence and stakeholder perspectives.

### 2.2. Team Formation and Stakeholder Engagement

The project was jointly led by JCDC and HCAC, who assembled a multidisciplinary team with designated roles, including project management, technical oversight, subject matter expertise, and administrative coordination. To ensure inclusivity and multisectoral representation, a stakeholder mapping exercise was conducted. The stakeholder mapping exercise identified key players from a variety of sectors as demonstrated in the Supplementary File S1.

To guide the project and ensure alignment with national priorities and global standards, a technical committee was established. This committee included representatives from all major sectors involved in laboratory services. Two pivotal meetings were convened: an inception meeting to review the study protocol and tools, and a validation meeting to discuss preliminary findings and provide technical feedback.

### 2.3. Tools Development and Data Collection

To obtain an extensive understanding of Jordan’s laboratory landscape, data collection was divided into three separate methods:

* Desktop Review: A four-week desktop review was conducted in June 2023 to gather publicly available data from national authorities, including annual reports, official websites, strategic documents, and program guidelines. Supplementary sources from the WHO, EMPHNET, IOM, USAID were also reviewed. To explore communicable disease patterns in Jordan, a targeted literature search was performed using Google Scholar, complemented with PubMed and WHO IRIS databases, applying a broad set of keywords related to respiratory, gastrointestinal, vector-borne, parasitic, fungal, sexually transmitted infections, AMR and zoonotic diseases (such as brucellosis, rabies, avian influenza) to ensure comprehensive coverage. Inclusion criteria were: studies conducted in Jordan between 2000 and 2023, published in English, full-text available, and of original or review type; case reports and editorials were excluded. In total, 226 documents were reviewed, representing a comprehensive range of governmental, non-governmental, and international sources.

* Key Informant Interviews (KIIs): The key informant interview process followed a structured methodology beginning with the identification of stakeholders across sectors involved in the national laboratory response to communicable diseases. Through collaboration between JCDC and HCAC, 20 key informants were selected and interviewed across 17 sessions in July 2023, representing public, private, military, and academic sectors. A semi-structured interview guide developed by HCAC and approved by JCDC guided the discussions, covering eight core sections including laboratory legislations, policies, SOPs, compliance, challenges, collaboration, future directions, and a closing section (Supplementary File S2). Interviews were conducted either virtually or face-to-face, with informed consent obtained and confidentiality assured. All data were transcribed verbatim and independently coded by two researchers, with any discrepancies resolved through consensus to ensure reliability. Thematic analysis was then carried out through a systematic approach that involved familiarization, categorization, and interpretation. Key themes such as regulatory frameworks, structural aspects, SOPs, integration of One Health, challenges, and recommendations were identified and synthesized.

* Field Visits: At the start of the project, technical teams from JCDC and HCAC jointly developed and approved a targeted list of laboratories for field visits. Laboratories were purposively selected to ensure broad representation across One Health stakeholders and geographic regions (Central, Northern, and Southern Jordan), including Ministry of Health’s (MoH) central and peripheral labs, Ministry of Agriculture (MoA)’s veterinary labs, private hospital and diagnostic labs, academic institutions, and NGO-affiliated facilities. Selection prioritized facilities with either comprehensive diagnostic services or specialized testing capacity for priority pathogens. The goal was to assess the overall capacity and functionality of the national laboratory network in responding to communicable diseases, rather than evaluating individual laboratories in isolation.

A structured questionnaire was designed to serve as the primary assessment tool during the field visits (Supplementary File S3). Framed within the One Health approach, the tool aimed to capture detailed insights into the current laboratory response system’s gaps, challenges, and operational limitations in relation to communicable diseases. The tool comprised four main sections: general information about the laboratory, governance and leadership, technical operations, and a specialized section for microbiology laboratories. Additionally, the technical operations section addressed areas such as national guidelines, communicable disease testing, workflow processes, standard operating procedures, quality control measures, testing algorithms, biosafety, and security. The microbiology section specifically focused on culture and sensitivity testing, media preparation, bacterial identification, and the laboratory’s capacity to detect high-priority pathogens. The field visits were initiated by formally reaching out to selected sites to obtain approvals and schedule appointments. Between 12 August and 20 September 2023, a total of 23 visits were conducted across 27 laboratories located in four governorates, namely, Amman, Irbid, Aqaba, and Karak. The selected laboratories represented a diverse range of One Health stakeholders, including governmental, private, military, academic, and NGO sectors. Supplementary Files S4 and S5 depict more details of the labs involved in the field visits.

### 2.4. Analysis and Evaluation

**Figure 1:**
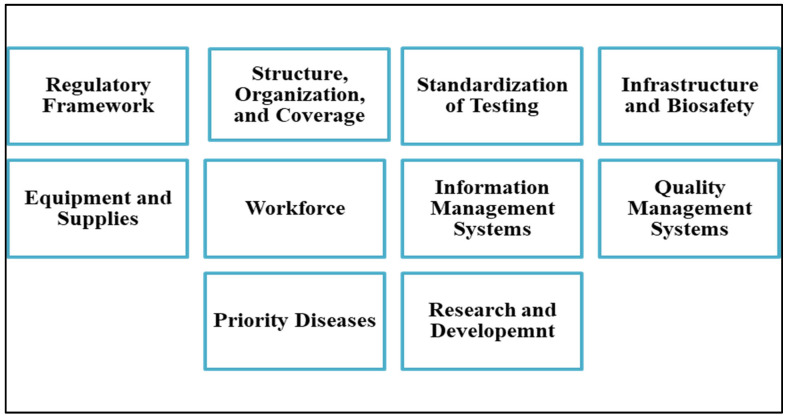
Laboratory Core Capacity- Adapted from the LABNET Score Card.

The findings from the multi-method approach, comprising the desktop review, KIIs, and field visits, were synthesized and organized according to ten core laboratory capacities and components adapted from the LABNET model developed by Ondoa et al. (2016) [16], which was originally designed to evaluate national laboratory networks in Africa in the context of global health security. In this model, core capacities represent the essential functions of a national laboratory network for detecting, assessing, reporting, and responding to public health threats within a One Health framework. These capacities align with international standards and guidance, including International Health Regulations (IHR), the Global Health Security Agenda (GHSA), the WHO guidance for establishing national health laboratory systems, the WHO Integrated Disease Surveillance and Response, the WHO global strategy for combating AMR, and the WHO regional guide for establishing laboratory-based surveillance of AMR. Figure 1 and Table 1 illustrate more details.

### 2.5. Validation Workshop and Reporting

A full-day validation workshop was held with 90 participants, including members of the technical committee and key stakeholders, to review and discuss the initial draft of the assessment. The feedback from the validation workshop was systematically incorporated into the final analysis; for example, participants highlighted gaps in biosafety guidelines and zoonotic disease diagnostics, which were subsequently emphasized in the results and recommendations.

### 2.6. Ethical Considerations

This study was conducted under the auspices of the JCDC. The Research, Health Policy, and Training Directorate reviewed the study protocol and concluded that formal ethical approval was not required, as the work did not entail patient involvement, animal experimentation, or the collection of identifiable health data. Instead, the study relied on secondary analysis of existing reports, alongside structured input from stakeholders. Accordingly, it received an exemption from full Institutional Review Board (IRB) review. Informed consent was obtained from all experts and stakeholders who participated in interviews and site visits. All procedures were conducted in strict alignment with the ethical principles articulated in the 1964 Declaration of Helsinki and its subsequent amendments.

## 3. Results

### 3.1. Policy and Regulatory Landscape

The policy framework for laboratory services in Jordan is only partially developed and outdated. No National Laboratory Strategic Plan (NLSP) or unified policy document exists, leaving the system without a consolidated vision. Governance instead relies on a patchwork of laws and ministerial instructions, many of which are outdated or limited in scope (See Table 2).

Laboratory responsibilities are dispersed across multiple authorities: the MoH oversees the Central Public Health Laboratory (CPHL) and hospital laboratories, the MoA manages veterinary laboratories, and additional capacity is provided by the RMS, academic institutions, and the private sector. However, no national coordination mechanism integrates these sectors. Oversight is fragmented, collaboration occurs mainly on an ad hoc basis during outbreaks, and no inter-sectoral body standardizes protocols or ensures data sharing between human and animal health laboratories. This lack of coordinated governance highlights the urgent need for a unified national approach.

Existing laboratory legislation is incomplete and outdated in key areas of quality and biosafety. The Public Health Law No. 47 (2008) provides the legal foundation for communicable disease reporting and implicitly recognizes zoonotic transmission, but it has not been updated to mandate modern One Health collaboration or enforce laboratory quality standards.

The Licensing System for Private Medical Laboratories (2003; amended 2018) sets basic requirements such as staff qualifications and equipment standards, but applies uniform criteria to all facilities regardless of risk or testing complexity. This approach fails to distinguish between small clinic laboratories and national reference centers. Enforcement is also weak, as licensing does not require accreditation or participation in proficiency testing. Together, these gaps highlight the need for risk-based and enforceable regulation to strengthen laboratory quality assurance.

Also, the only formal national guidance on laboratory quality is the MoH Internal Quality Control (IQC) Instructions from 2006. These have never been revised and are not aligned with international standards, limiting their relevance to current practice.

Guidelines for transporting infectious substances exist but do not comply with international standards (IATA/ICAO), lacking requirements for certified packaging, labeling, and shipper training.

Biosafety and biosecurity regulations are underdeveloped. Jordan has no comprehensive biosafety law, and existing guidelines—mainly internal protocols—are inconsistent and unevenly enforced. National provisions for occupational health cover general safety, but specific standards for handling high-risk pathogens (e.g., BSL-3 laboratories) are absent.

In summary, the policy landscape for laboratories shows a foundational legal structure for communicable disease control and laboratory licensing, but with significant gaps in strategic planning, quality governance, and inter-sectoral coordination. Key regulations exist but are outdated and do not fully meet international standards or the current needs of Jordan’s public health laboratory network. This context of fragmented oversight and dated policies provides the background for the more technical findings on laboratory operations and capacities described below.

### 3.2. Availability of Laboratory SOPs and Guidelines for Communicable Diseases

Our study revealed uneven availability of SOPs and laboratory guidelines across diseases. Conditions with strong vertical programs or external support had well-defined protocols, while many others lacked them.

For high-priority diseases, several structured SOPs exist. The National Tuberculosis Program maintains a comprehensive manual covering microscopy, culture, and drug susceptibility testing, last updated within the past decade and aligned with WHO standards. The national HIV program has a testing algorithm that guides both screening and confirmatory testing. The National Influenza Center at the CPHL implements WHO protocols for influenza surveillance and PCR testing. During the COVID-19 pandemic, emergency guidelines for SARS-CoV-2 diagnostics were rapidly developed and subsequently institutionalized, becoming the routine SOPs for PCR testing and biosafety. Polio testing also follows WHO laboratory network protocols, with stool specimens referred to a regional reference lab.

In contrast, zoonotic and less common infections lacked national SOPs. Brucellosis testing is performed using culture and serology, but without a unified national guideline. Rabies diagnostics remain a major gap: no human laboratory confirmation is available (cases are diagnosed clinically or samples sent abroad), and veterinary laboratories have no capacity or SOPs for rabies testing. Other diseases, such as hemorrhagic fevers (e.g., CCHF), also lack dedicated SOPs, with labs depending on ad hoc instructions or international reference laboratories when needed. Foodborne pathogens such as Salmonella are diagnosed using ISO or WHO standard methods, but no national outbreak-specific laboratory guideline exists.

Even where SOPs exist, their quality and consistency vary. Some are outdated or exist only in draft form—for example, a draft “Pandemic Diseases Laboratory Guide” was never officially adopted. Larger facilities like the CPHL and major hospital laboratories often maintain internal SOP compilations for routine tests, whereas smaller regional or peripheral laboratories frequently lack formal documentation beyond basic bench methods. This creates variability in practice across the network. Table 3 highlights representative examples, showing strong SOP coverage for TB, HIV, influenza, COVID-19, and polio, but significant gaps for zoonoses, rabies, hemorrhagic fevers, and foodborne pathogens. In this table, the designation “Yes—Present” indicates the existence of an official or widely adopted SOP or guideline, while “No” denotes the absence of a dedicated national laboratory protocol for the respective disease. Overall, Jordan’s laboratory system demonstrates the capacity to develop and implement SOPs for selected high-priority diseases, especially when supported by international programs. However, the absence of comprehensive and regularly updated SOPs for many other communicable diseases leaves laboratories reliant on general methods or external manuals, contributing to inconsistent practices and reduced preparedness for emerging infections.

### 3.3. Quality Assurance and Laboratory Quality Management

The assessment found that Jordan’s quality management system is nascent and unevenly applied. Internal quality control (IQC) is practiced in most laboratories, but the only national guidance is the MoH’s 2006 QC instructions, which are outdated and not aligned with ISO 15189. As a result, implementation varies widely: larger facilities such as the CPHL and major hospitals follow systematic IQC procedures, while peripheral laboratories often rely on informal practices.

Participation in external quality assurance (EQA) is limited to a few programs. The TB laboratory at CPHL takes part in WHO supranational proficiency testing, the National Influenza Center joins WHO EQA panels, and HIV testing has occasionally been validated through international schemes (e.g., CDC/WHO). Outside these programs, routine EQA is rare; most bacteriology, serology, and parasitology labs lack proficiency testing, and no national EQA scheme exists to provide system-wide oversight. Consequently, errors and variability in testing may go undetected until problems arise.

Accreditation opportunities exist but coverage is uneven. National accreditation bodies such as HCAC and JAS, as well as international frameworks (ISO 15189, ISO 17025, CAP, UKAS, DAKKS), are available. Over 70 laboratories across sectors have achieved accreditation, including three MoH medical laboratories, and several others are in progress. However, accreditation remains concentrated in select facilities, while most sectoral and peripheral labs still operate without recognized quality systems.

A major gap is the absence of a national QA coordination unit or reference center. Quality activities are left to individual laboratories or programs, with no unified system to distribute EQA panels, monitor performance, or require compliance. This lack of central coordination prevents quality management from being embedded into licensing or regulatory enforcement.

Biosafety and biosecurity practices are basic but inconsistent. Most labs use PPE and biosafety cabinets where available, and staff are commonly vaccinated against Hepatitis B. However, Jordan lacks a comprehensive biosafety law or updated national guidelines. Biosafety Level 3 capacity is very limited (restricted mainly to TB culture at CPHL), and veterinary laboratories lack such facilities. Biosecurity controls such as pathogen inventory management and access restrictions are rudimentary outside of top-tier labs. Concerns during the COVID-19 surge highlighted the risks of rapid expansion of diagnostic testing without uniform biosafety training or waste management systems.

Despite these weaknesses, there are positive practices to build on. Donor-funded initiatives have supported training in laboratory methods, infection prevention and control, and surveillance. Yet advanced skills remain scarce in molecular diagnostics, bioinformatics, equipment calibration and maintenance, and quality system management. Table 4 summarizes the status of key quality assurance components in Jordan’s laboratory system as identified by this study.

The findings on quality assurance highlight critical weaknesses that could compromise diagnostic reliability. Without consistent EQA and accreditation, there is a risk of undetected errors or variability in test performance across different laboratories. The lack of a formal quality management framework means improvements rely on individual initiative rather than a mandated standard. These results underline the need for a concerted effort to establish a robust QA system—including updating guidelines, instituting regular proficiency testing, and moving toward accreditation of laboratories or key tests to ensure high and uniform performance.

### 3.4. Workforce Capacity and Training

Jordan benefits from a cadre of skilled laboratory professionals: most MoH and sectoral laboratories are staffed by qualified technicians, technologists, and specialists with bachelor’s or higher degrees. Technical capacity is strongest at the CPHL and central hospitals, where staff routinely perform microbiological culture, antimicrobial susceptibility testing, and molecular diagnostics such as PCR. Many laboratorians are familiar with WHO/CDC protocols, have strong language skills, and some have received international training. This foundation enabled rapid scale-up of COVID-19 testing capacity when resources became available.

At the same time, the workforce is stretched thin and unevenly distributed. Peripheral and veterinary laboratories face pronounced shortages, and staffing is barely sufficient for routine services, leaving little surge capacity for emergencies. The COVID-19 pandemic illustrated this vulnerability, as MoH laboratories quickly became overwhelmed and testing had to be outsourced. Turnover is high, with experienced staff frequently moving to the private sector or abroad due to better salaries, resulting in loss of institutional knowledge.

Training and professional development opportunities are limited. Most staff have not received refresher training in new diagnostic technologies, quality management, or biosafety in recent years. While WHO- and donor-supported workshops are offered intermittently, there is no structured continuing education system or clear career path-way. Specialized skills such as molecular diagnostics, bioinformatics, and quality assurance are underrepresented. The laboratory track in Jordan’s Field Epidemiology Training Program provides some exposure, but involves only a small number of participants annually.

Academic institutions continue to produce graduates in laboratory sciences, yet many require additional hands-on training to meet public health needs, and retention in the public sector is low. Despite these constraints, commitment among existing staff remains strong, with many laboratorians multitasking to cover gaps and demonstrating adaptability during crises.

**Figure 2:**
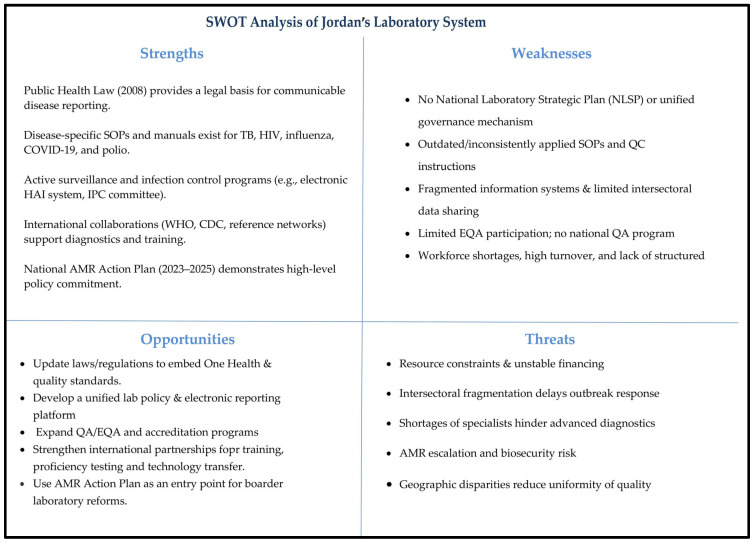
SWOT analysis of Jordan’s laboratory system for communicable diseases.

In summary, Jordan’s workforce is well trained at baseline but insufficient in number, poorly retained, and under-supported by structured professional development. Targeted recruitment, retention incentives, and continuous training are critical to sustain and expand laboratory capacity. To consolidate the findings, a SWOT analysis as shown in Figure 2 was conducted to provide a structured overview of the internal and external factors shaping Jordan’s laboratory system for communicable diseases. This approach highlights strengths and weaknesses within the system, while also identifying opportunities for improvement and external threats that could hinder progress. Presenting these elements in a single framework allows stakeholders to clearly visualize priority areas for action, inform strategic planning, and align laboratory strengthening efforts with international health security and One Health objectives.

## 4. Discussion

This study represents the first comprehensive national mapping of Jordan’s laboratory system for communicable diseases, using the LABNET framework to evaluate policies, legislation, SOPs, and operational practices. By consolidating fragmented information across human and animal health laboratories, the analysis offers a system-wide overview that highlights both achievements and persistent gaps. The findings confirm that while Jordan has made important progress—particularly in program-specific areas such as TB, HIV, influenza, and COVID-19 diagnostics—systemic weaknesses in governance, financing, quality assurance, biosafety, and workforce development remain unresolved.

In this section, we situate these findings within the broader context of Jordan’s public health infrastructure and international benchmarks, draws comparisons with similar regional and global experiences, and explores their implications for health security and One Health. In doing so, it addresses root causes identified during the mapping—namely limited horizontal governance, outdated regulatory frameworks, constrained financing, and fragmented intersectoral coordination—and outlines strategic opportunities to align Jordan’s laboratory system with international standards such as the IHR 2005 and the GHSA.

### 4.1. Contextualizing Jordan’s Laboratory System Within Public Health Infrastructure

Jordan’s laboratory network operates within a broader public health system that has demonstrated both resilience and fragility. The MoH, through the CPHL, serves as the reference center, supporting governorate and hospital laboratories by providing confirmatory testing, such as for multidrug-resistant isolates [6,11]. In the 2016 Joint External Evaluation (JEE), Jordan’s diagnostic capacity for priority diseases was rated as “demonstrated” (score 3/5), reflecting the ability to perform confirmatory testing and maintain specimen referral systems [17]. However, the JEE also highlighted weaknesses in quality systems, governance, and biosafety, which remain unresolved.

Jordan’s geographic location and humanitarian context present unique pressures. High cross-border mobility, large refugee populations, and recurring regional outbreaks elevate risks of importation and spread of communicable diseases [1,4,18]. The COVID-19 pandemic underscored both strengths and vulnerabilities: while Jordan successfully expanded molecular testing through MoH–private sector collaboration [11], the surge exposed gaps in surge staffing, biosafety enforcement, and laboratory data integration [4,18].

These findings align with international evidence that laboratories are the backbone of communicable disease detection and response [5,6,19,20]. Accurate diagnostics underpin timely public health action, and system fragility in quality assurance or governance can compromise outbreak preparedness. By mapping Jordan’s laboratory-related legislation, policies, and SOPs, this study provides a critical baseline for strengthening integration with public health infrastructure and guiding modernization to align with international standards.

### 4.2. Alignment with International Standards and Global Benchmarks

Situating Jordan’s laboratory capacity within the benchmarks of the IHR 2005 and GHSA provides an important perspective on both progress and persisting gaps. Under the IHR, laboratory services are a core capacity for detecting, assessing, reporting, and responding to public health risks [20]. In Jordan’s 2016 JEE, diagnostic testing for priority diseases and specimen referral systems were scored as “demonstrated capacity” [17]. These strengths highlight a functional baseline, particularly in clinical and program-specific laboratories.

However, the same JEE identified quality management, biosafety, and governance as limited, scoring only 2/5 in laboratory quality systems [17]. This reflects the absence of a comprehensive NLSP, which international frameworks emphasize as critical for coordinating investments and setting a unified vision [21]. While Jordan has achieved notable progress in test-specific accreditation (e.g., ISO 15189, CAP, HCAC), these remain fragmented and largely voluntary [22]. Global guidance stresses that a national laboratory policy defines the vision of the laboratory system, and a strategic plan operationalizes it; the absence of such instruments leaves Jordan without a roadmap for sustainable system-wide improvement [21,23].

Outdated national regulations further limit compliance with international standards. For example, the 2006 MoH QC instructions are not aligned with ISO 15189 requirements, and national guidelines for infectious substance transport lack mandatory provisions from IATA/ICAO [22]. The Public Health Law No. 47 of 2008 provides a strong foundation for communicable disease reporting but does not explicitly mandate One Health coordination or laboratory quality enforcement, contrary to best practice in countries that have updated legislation to support multisectoral surveillance [21].

Compared to global trends, Jordan mirrors many middle-income countries where strong vertical disease programs coexist with fragmented oversight. Sub-Saharan African countries, for instance, have faced similar challenges—weak financing, incomplete quality systems, and disconnects between policies and implementation—until coordinated reforms were implemented [21]. These comparisons underline that while Jordan is not unique in its challenges, adopting an NLSP and updating regulatory frameworks would align the system with international best practices and accelerate progress toward IHR compliance.

In addition, the GHSA emphasizes laboratories as a cornerstone of national health security, with explicit targets for quality management, biosafety, and data integration [20]. Jordan’s recent participation in internationally supported initiatives, including the FAO/WHO/UNICEF Pandemic Readiness Enhancement Program [24], provides an opportunity to modernize laboratory infrastructure, implement standardized protocols, and institutionalize quality frameworks in line with IHR and GHSA priorities.

### 4.3. Gaps, Strengths, and Implications for Health Security and One Health

The findings revealed that Jordan’s laboratory system combines established technical capacity with persistent systemic gaps that undermine resilience. On the positive side, the country benefits from an established legal foundation through the Public Health Law No. 47 (2008), functional specimen referral pathways, and skilled professionals capable of supporting priority disease programs such as TB, HIV, influenza, and COVID-19 [17,22]. Jordan also participates in international surveillance and reporting mechanisms, including WHO’s influenza network and food safety networks, which provide external validation and technical support [19,24].

A key finding of this study is the uneven distribution of disease-specific SOPs across the laboratory network. Updated SOPs exist for T.B, HIV, influenza, and COVID-19, often supported by WHO or program-specific guidance, enabling accurate and standardized diagnostics. However, other notifiable diseases, particularly zoonoses such as brucellosis and rabies, as well as foodborne pathogens and hemorrhagic fevers, lack formal SOPs, with laboratories relying on general microbiological methods or international manuals [12,14]. This inconsistency undermines preparedness for zoonotic and emerging infections. Strengthening laboratory SOPs for priority zoonoses under a One Health framework, coupled with harmonized quality assurance and biosafety measures, will be essential for building resilience and aligning Jordan with international standards.

Yet, critical gaps persist. Laboratories across sectors remain fragmented, with no empowered national coordinating mechanism to unify policies, quality assurance, or biosafety standards. This fragmentation mirrors findings from other LMICs, where absence of clear governance structures has been identified as a root cause of underperformance [21]. In Jordan, weak horizontal governance, outdated licensing regulations, and the lack of a national external quality assurance (EQA) program reflect institutional design failures rather than technical capacity alone [21,22].

Financing constraints exacerbate these challenges. Limited and no earmarked funding for QA/EQA and biosafety activities has impeded sustainability, while rigid civil service pay structures constrain retention of skilled laboratorians, particularly in advanced molecular diagnostics and quality management [21]. The COVID-19 pandemic exposed these vulnerabilities, as surge capacity was achieved only through temporary hiring and private sector outsourcing [11].

These systemic weaknesses carry major implications for health security. Laboratory quality gaps increase the risk of false negatives or positives, undermining trust in diagnostic results and delaying outbreak detection. Limited biosafety oversight raises the potential for laboratory-acquired infections or accidental releases. Equally important, the lack of routine coordination with animal health laboratories impedes early warning for zoonotic threats. Jordan’s inability to conduct in-country confirmation of rabies in humans or animals illustrates this challenge [12]. Such gaps leave the country vulnerable to zoonotic spillover, which accounts for an estimated 60–75% of emerging infectious diseases globally [25].

At the same time, the study highlighted significant strengths that can be leveraged. Jordan’s laboratory professionals have demonstrated adaptability and commitment, particularly during the COVID-19 pandemic, when testing capacity was rapidly scaled up [11]. International collaborations—such as WHO EQA schemes for influenza and TB—demonstrate that external quality participation is feasible when institutionalized. Furthermore, the government has expressed growing recognition of the One Health approach, reflected in draft frameworks and joint trainings for zoonoses such as avian influenza and rabies [26].

In summary, Jordan’s laboratory system has both a foundation to build on and structural barriers that require reform. Root causes lie less in technical knowhow and more in governance, financing, and workforce support. Addressing these will be essential to ensuring reliable diagnostics, strengthening health security, and operationalizing One Health in practice.

### 4.4. Regional and International Comparision

Jordan’s laboratory system challenges mirror patterns seen in many middle-income countries: robust vertical disease programs but fragmented governance, uneven quality systems, and incomplete One Health integration. Comparative experience from sub-Saharan Africa shows that these deficits are tractable when countries adopt a national laboratory strategic plan, institute coordinating bodies, and roll out stepwise quality improvement (e.g., SLIPTA/SLMTA) [20,21]. Cameroon’s IHR–PVS National Bridging Workshop (NBW), for instance, produced a jointly owned One Health roadmap that fed directly into its National Action Plan for Health Security, formalizing cross-sector coordination and joint activities [27].

Within the EMR, WHO/EMRO’s Operational Framework for One Health (2022–2027) provides a region-specific playbook that emphasizes governance platforms, joint risk assessment (JRA), interoperable surveillance/lab data flows, and biosafety/biosecurity alignment. This framework was reinforced by the Quadripartite regional meeting (Muscat, May 2023) that prioritized operationalization of multi-sector coordination for zoonoses and AMR across EMR health systems [26,28]. These EMR guidance documents are directly relevant to Jordan’s findings on weak horizontal governance, missing national EQA, and limited human–animal lab data sharing, and they align closely with the reforms we propose (national coordination mechanism, NLSP, and One Health data integration).

Country experiences in the Gulf illustrate practical One Health implementation that Jordan can adapt. In Qatar, an inter-ministerial One Health structure established since the MERS era has been used to strengthen joint surveillance and preparedness, culminating in a published national One Health framework with procedures for real-time cross-sector information exchange and joint risk analysis [29,30]. In Saudi Arabia, large-scale avian influenza surveillance integrated veterinary and public health laboratory networks—thousands of birds were tested across provinces with results informing national response—showcasing how networked veterinary–public health labs can deliver actionable early warning intelligence [31].

Closer to EMRO’s fragile context, Somalia’s 2024 National Bridging Workshop (NBW) demonstrated that even in resource-constrained environments, formal One Health coordination mechanisms and joint operational plans can be instituted through the NBW process—an operational model Jordan could replicate to institutionalize its cross-sector laboratory coordination and JRA cycles [32].

Taken together, African and EMR experiences converge on the same levers Jordan needs now: (i) a multisectoral coordination mechanism that owns and oversees laboratory strengthening; (ii) a National Laboratory Strategic Plan aligned with EMRO’s One Health operational framework; (iii) institutionalized EQA/accreditation pathways using stepwise models proven in Africa; and (iv) shared human–animal laboratory data systems with routine JRA—approaches that have demonstrably improved laboratory performance and health security outcomes in comparable settings [20,21,27,28,29,30,31,32].

### 4.5. Recommendations and Way Forward

The study confirmed that Jordan’s laboratory system has strong technical foundations in specific programs but remains constrained by systemic weaknesses in governance, outdated regulations, and limited quality assurance. These issues are not unique: similar gaps have been documented in other LMICs, where targeted reforms have proven successful. Based on our findings, and consistent with international frameworks such as the IHR (2005), GHSA, WHO/AFRO guidance, and APHL standards, a set of evidence-based recommendations is proposed.

1. Developing and implementing a National Laboratory Strategic Plan (NLSP).

The absence of an NLSP emerged as one of the most critical gaps. Without such a plan, laboratory activities across the MoH, MoA, Royal Medical Services, academia, and private sector remain fragmented and reactive. This gap was highlighted in Jordan’s 2016 JEE, which called for a national laboratory policy and strategic plan [17]. WHO and APHL emphasize that an NLSP provides the backbone for laboratory system strengthening [23,33]. Experiences from Uganda and Ethiopia demonstrate that national strategies, combined with SLMTA, accelerated accreditation and improved quality within a decade [20]. Jordan should prioritize the development of a multisectoral NLSP with a One Health focus, aligning investments and coordination across human, animal, and environmental health domains.

2. Updating and harmonizing the legal and regulatory framework.

Core legal instruments—including the Public Health Law No. 47 (2008), the Licensing System for Private Medical Laboratories, and the MoH QC guidelines (2006)—are outdated and inconsistent with modern standards. They lack provisions for accreditation, biosafety, or systematic EQA participation [22]. International best practices call for risk-based licensing and harmonized biosafety/quality requirements [21,26]. Countries such as Cameroon and Kenya revised their laws through IHR–PVS bridging workshops to institutionalize joint surveillance and One Health approaches [27]. Jordan should revise its laws to: (i) mandate QA/EQA participation, (ii) categorize laboratories by testing complexity and risk, and (iii) align biosafety and transport regulations with ISO 15189 and IATA/ICAO standards.

3. Establishing a national coordination mechanism.

Fragmentation across MoH, MoA, RMS, and the private sector remains a root cause of inefficiency. Our mapping found no empowered body to oversee laboratories or harmonize SOPs. Internationally, coordination mechanisms have proven transformative. In Cameroon and Kenya, bridging workshops led to the creation of national laboratory coordinating committees. WHO EMRO also explicitly calls for unified governance across sectors under a One Health framework [26]. Jordan should establish a National Laboratory Coordination Committee under the Higher National Committee for Epidemics or JCDC to oversee NLSP implementation, harmonize SOPs, and ensure efficient division of roles.

4. Strengthening quality assurance and workforce development.

Quality assurance practices in Jordan remain inconsistent, with no national EQA program and uneven accreditation coverage. This reflects a systemic gap also noted in the JEE [18]. International experience shows that stepwise programs such as SLIP-TA/SLMTA improve quality and accreditation outcomes [20,23]. Establishing a national EQA reference center, possibly hosted at JCDC, would provide proficiency panels and monitor system-wide performance. Workforce shortages and turnover further undermine laboratory capacity, as highlighted during COVID-19. Expanding Jordan’s FETP laboratory track, investing in continuous professional development, and improving retention incentives would mirror successful workforce strengthening models from AFENET [20].

5. Enhancing One Health surveillance and data sharing.

Weak integration between human and animal laboratory systems limits Jordan’s ability to detect zoonotic threats. Our mapping highlighted gaps in rabies and brucellosis diagnostics, where data are collected separately by MoH and MoA [12]. International evidence shows the value of integrated electronic platforms and joint risk assessments [26]. Jordan has piloted Joint Risk Assessments and is already participating in the FAO-led Pandemic Readiness Enhancement Program, which supports laboratory modernization and One Health integration [15]. Building on this momentum, Jordan should establish a shared One Health laboratory database and institutionalize joint simulations and cross-sector analyses. Models from Qatar (MERS-CoV) and Saudi Arabia (influenza) illustrate the benefits of integrated human–animal lab surveillance for early warning and rapid response.

The evidence-based recommendations and their alignment with global best practices underscore that addressing governance, legal, quality, and One Health integration is not optional but essential to health security. Implementing these reforms will not only enhance Jordan’s compliance with international obligations under the IHR and GHSA but also position the country as a regional leader in laboratory system strengthening. The synthesis of national findings with international evidence ensures that the proposed actions are both context-specific and globally relevant, offering a practical roadmap for decision-makers and stakeholders. Please see Table 5 for more details. Together, these recommendations respond directly to the gaps identified in this study. They are grounded in Jordan’s context but consistent with internationally endorsed approaches, ensuring that reforms are evidence-driven and aligned with IHR and GHSA capacities.

Our study has several limitations that should be acknowledged. The selection of laboratories for field visits was purposive rather than random, prioritizing facilities with essential functions across the human and animal health sectors. These included laboratories from military, governmental, academic, private, and non-governmental organization sectors, distributed across the three regions of Jordan (central, north, and south). While this ensured broad representation across One Health stakeholders, it may limit the representativeness of the findings for all laboratories nationwide. Second, the desktop review relied primarily on available national legislation, guidelines, and program documents, supplemented with international sources. Some unpublished or internal materials may not have been accessible, which could affect the completeness of the document mapping. Third, findings from key informant interviews reflect the perspectives of the participants and are subject to potential recall or perception bias; however, triangulation with document review and field observations, combined with consensus validation, mitigated this risk. Fourth, the assessment provides a time-bound snapshot of Jordan’s laboratory system as of 2023. Rapid policy or programmatic changes that have occurred since may not be captured in this analysis. Finally, as this was a country-specific assessment, the findings are not intended to be directly generalizable to other settings, although the use of the internationally recognized LABNET framework enhances transferability of lessons learned. Despite these limitations, the multi-method design and the national validation workshop strengthened the robustness of the results, ensuring that the conclusions are credible and policy-relevant.

## 5. Conclusions

This study provides the first comprehensive mapping of Jordan’s laboratory system for communicable diseases, applying the LABNET framework to evaluate legislation, policies, SOPs, and operational practices. The analysis revealed that while Jordan has achieved important progress—particularly in program-specific areas such as TB, HIV, influenza, and COVID-19 diagnostics—systemic gaps in governance, financing, quality assurance, biosafety, and workforce development continue to constrain overall performance.

By aligning the findings with international standards and regional experiences, the study highlights both the urgency and feasibility of reforms. Evidence-based recommendations were developed to address the identified gaps, focusing on the development of a National Laboratory Strategic Plan, updating legal frameworks, establishing a coordination mechanism, strengthening quality assurance and workforce development, and advancing One Health surveillance. These measures are grounded in best practice and international guidance, ensuring that reforms are practical and scalable.

Implementing these actions will enhance Jordan’s compliance with the IHR 2005, accelerate progress under the GHSA, and improve preparedness for emerging infectious diseases. More broadly, a strengthened and coordinated laboratory system will reinforce Jordan’s role as a regional leader in advancing health security and One Health integration.

## Figures and Tables

**Table 1:** Core Capacities and Components considered for Evaluating the National Laboratory Network.

No.	Core Capacity	Sub Components
1	Political, Legal, Regulatory and Financial Framework	Legislation Governance National policies and plans Finances
2	Structure, Organization, and Coverage	Tiered laboratory network Coverage Multisectoral Collaboration
3	Standardization of Testing	Guidelines and protocols Diagnostic algorithms
4	Infrastructure and Biosafety	Infrastructure Biosafety and Biosecurity
5	Equipment and Supplies	Equipment Supply Chain Management
6	Workforce	Workforce Human Resource Strategies and Management Education and Training
7	Information Management Systems	Surveillance and Epidemiology Reporting
8	Quality Management System	Quality Assurance (QC, and EQA) Certification and Accreditation
9	Priority Diseases	Prioritization Antimicrobial resistance
10	Research	Research and Development

**Table 2:** Key Laboratory-Related Policies and Regulations in Jordan.

Policy/Regulation	Year	Scope and Coverage	Observations from Mapping
Public Health Law No. 47 (Chapter V)	2008	Communicable disease reporting, outbreak response, and some One Health principles (human–animal health)	Provides legal basis for disease notification. Not updated since 2008; does not explicitly mandate lab quality standards or modern One Health coordination.
Licensing of Private Medical Laboratories Regulation	2003 (amended 2018)	Licensure requirements for private clinical laboratories (staff qualifications, equipment, safety, etc.)	Applies uniform criteria to all private labs. Lacks risk-based categorization of labs by testing complexity. Limited provisions for ongoing quality monitoring (no mandatory proficiency testing or accreditation).
MoH Internal Quality Control Instructions	2006	Guidelines for internal quality control procedures in medical laboratories.	Outdated—not aligned with current quality management best practices (e.g., ISO 15189). No updates or enforcement mechanism; implementation varies by lab.
Infectious Substance Transport Guidelines	N/A (in use)	Instructions for packaging and transporting infectious samples between labs (domestic referrals).	Not aligned with IATA/ICAO international transport standards. Missing detailed requirements for certified packaging, labeling, and shipper training. Poses compliance and safety risks.
Biosafety/Biosecurity Guidelines	N/A (various)	Basic lab safety rules and biohazard handling procedures (internal to MoH and some institutes).	No unified biosafety laws. Existing guidelines are fragmented and not consistently enforced. Biosafety level standards (e.g., for BSL-3) not formally adopted at national level.
National Laboratory Policy/Strategy	Not present	(Would outline national vision, roles, and development plans for labs)	No official national lab policy or strategic plan exists, leaving a strategic gap in laboratory system development.

**Table 3:** Availability of Laboratory SOPs/Guidelines for Selected Communicable Diseases in Jordan.

Disease/Condition	Laboratory SOP/Guideline Availability	Notes (Source and Last Update)
Tuberculosis (TB)	Yes—Present. National TB Laboratory Manual available.	NTP lab manual in place (updated ~2018; aligned with WHO TB guidelines). Widely used in MoH TB labs.
HIV/AIDS	Yes—Present. HIV testing algorithm and guidelines.	National HIV testing guidelines (covering ELISA/rapid testing and confirmatory strategies). Updated in recent years via National AIDS Program.
Influenza (seasonal and avian)	Yes—Present. SOPs for sample collection and PCR testing.	Implemented at CPHL/National Influenza Center. Follows WHO protocols for influenza surveillance; includes avian influenza testing procedures.
COVID-19	Yes—Present. SARS-CoV-2 testing SOPs (PCR).	Developed in 2020; incorporated into MoH lab practices. Includes specimen handling, PCR protocols, and safety measures.
Poliomyelitis	Yes—Present. Polio laboratory testing protocol.	Jordan uses WHO polio laboratory network protocols. Stool specimen referral to regional reference lab; national lab follows standard WHO polio SOPs for sample handling.
Brucellosis	No dedicated SOP. Uses general microbiological methods.	Culture and serological tests performed at central labs, but no single national guideline document; labs refer to WHO/FAO manuals as needed.
Rabies (Human)	No in-country testing SOP. Clinical diagnosis; samples sent abroad.	No routine lab confirmation for human rabies in Jordan. Suspect cases managed clinically or confirmed via external reference (e.g., CDC) if at all.
Rabies (Animal)	No capacity/SOP. Not performed domestically.	Veterinary labs lack facility for rabies testing (no fluorescence antibody test in-country). Suspected animal cases are euthanized without lab confirmation.
Hemorrhagic Fevers (e.g., CCHF)	No dedicated SOP. Ad hoc approach for diagnostics.	Rare cases would rely on external reference lab testing or use of WHO guidelines. No local SOP due to infrequent occurrence.
Foodborne Pathogens (e.g., Salmonella)	Partial. Standard lab methods followed; no national guideline.	Public health labs follow ISO or WHO standard methods for culture and identification of common foodborne bacteria, but no unified national document specific to foodborne outbreak lab response.

**Table 4:** Quality Management and Assurance Practices in Jordan’s Communicable Disease Laboratories.

Quality Component	Status in Jordan	Comments
Internal Quality Control (IQC)	Practiced in most labs, but no updated national guidelines or standardization.	Basic IQC (controls with tests) is common, especially in larger labs. However, the only official guidance is the 2006 instruction, which is outdated. Smaller labs have variable compliance.
External Quality Assurance (Proficiency Testing)	Limited participation; not systematized or mandatory.	Only specific programs (TB, HIV, influenza, etc.) engage in EQA via international networks. Majority of labs do not undergo regular proficiency testing. No national EQA program or requirement in place.
Laboratory-specific, non-laboratory-specific, animal, environmental and food safety accreditation standards	Examples on Laboratory-specific accreditations: College of American Pathologists (CAP) HCAC medical laboratories accreditation International Organization for Standardization (ISO) 15189:2003 Examples on Non-laboratory-specific accreditations: HCAC hospital accreditation Joint Commission International (JCI) hospital accreditation DAKKS Accreditation (International) UKAS Accreditation (International) ISO 9001 ISO 27001 Animal health, environmental health, and food safety ISO 17025	
Quality Management System (QMS)	Absent or ad hoc in most labs. Quality activities not formally coordinated.	No lab has a complete QMS according to ISO or SLIPTA framework. However, Jordan offers several laboratory accreditations programs at both national and international levels, including: JAS Accreditation (National) HCAC Accreditation (National) DAKKS Accreditation (International) UKAS Accreditation (International) ISO 15189:2003 (International) CAP Accreditation (International) Quality managers are not designated in most labs. Any QMS elements (SOPs, document control, audits) are implemented locally in an inconsistent manner.
National QA Coordination	Not established. No central QA unit or oversight body for lab quality.	Each lab or program handles quality on its own. There is no national reference laboratory or committee sending EQA panels or monitoring quality across labs.
Biosafety/Biosecurity	Basic measures in place; lacks comprehensive standards and enforcement.	Labs use PPE and biosafety cabinets where available. No updated biosafety guidelines or training program nationally. BSL-3 capacity very limited (only at CPHL for TB). Biosecurity controls are minimal outside top-tier labs.

**Table 5:** Evidence-Based Recommendations for Strengthening Jordan’s Laboratory System.

Domain	Key Gap (Findings)	Recommendation	Supporting Evidence/International Best Practice
Strategic Planning	No NLSP; fragmented vision across sectors	Develop and implement an NLSP with One Health focus	WHO and APHL guidance [23,33]; Uganda/Ethiopia experience with SLMTA and ISO accreditation [20]
Legal and Regulatory Framework	Outdated Public Health Law (2008); uniform licensing; outdated QC (2006)	Revise laws to mandate QA/EQA, risk-based licensing, and updated biosafety/transport standards	WHO EMRO One Health framework [26]; IHR–PVS bridging workshops (Cameroon, Kenya) [27]
Governance and Coordination	No empowered national coordinating mechanism; siloed MoH/MoA/private labs	Establish a National Laboratory Coordination Committee under JCDC/Epidemics Committee	WHO EMRO unified governance [26]; Cameroon/Kenya coordinating committees [27]
Quality Assurance and Workforce	No national EQA program; uneven accreditation; workforce shortages and turnover	Establish EQA reference center; adopt SLIPTA/SLMTA; expand FETP-lab track; strengthen CPD and incentives	WHO/AFRO stepwise quality improvement [20,23]; AFENET workforce strengthening
One Health Surveillance	Weak human–animal lab integration; no unified zoonotic reporting	Create shared One Health laboratory database; institutionalize JRAs and simulation exercises	FAO Pandemic Readiness Programme (Jordan) [24]; regional models (Qatar, Saudi Arabia) [28,29,30,31]

## Data Availability

No new data were generated in this study.

## Supplementary Materials

The following supporting information can be downloaded at: https://www.mdpi.com/article/10.3390/ijerph22091459/s1. Supplementary File S1: List of Key Stakeholders; Supplementary File S2: Semi Structured Interview Guide; Supplementary File S3: Field Visit Tool. Supplementary File S4: General Information on Participating Labs; Supplementary File S5: Conducted Field Visits.

## Abbreviations

AMR	Antimicrobial Resistance
BSL	Biosafety Level
CPHL	Central Public Health Laboratory
COVID-19	Coronavirus Disease of 2019
DAKKS	Deutsche Akkreditierungsstelle (German Accreditation)
EMPHNET	The Eastern Mediterranean Public Health Network
EQC	External Quality Control
FAO	Food and Agriculture Organization of the United Nations
HIV	Human Immunodeficiency Virus
HCAC	Health Care Accreditation Council
IATA	International Air Transport Association
ICAO	International Civil Aviation Organization
IHR	International Health Regulations
IOM	International Organization for Migration
IQC	Internal Quality Control
JCDC	Jordan Center for Disease Control
JEE	Joint External Evaluation
JFDA	Jordan Food and Drug Administration
KII	Key Informant Interview
MoA	Ministry of Agriculture
MoEnv	Ministry of Environment
MoH	Ministry of Health
NGO	Non-Governmental Organization
NLSP	National Laboratory Strategic Plan
PPE	Personal Protective Equipment
PCR	Polymerase Chain Reaction
QMS	Quality Management System
QC	Quality Control
RMS	Royal Medical Services
SOP	Standard Operating Procedure
TB	Tuberculosis
USAID	United States Agency for International Development
WAJ	Water Authority of Jordan
WEEC	Water, Energy and Environment Center
WHO	World Health Organization
CDC	U.S Centers for Disease Control and Prevention
GHSA	Global Health Security Agenda
LMICs	Low- and Middle-Income Countries
FDA	U.S Food and Drug Administration
NHS	National Health Service
IOS	International Organization for Standardization
UNICEF	United Nations Children’s Fund
UKAS	The United Kingdom Accreditation Service
WHO EMRO	The WHO Regional Office for the Eastern Mediterranean
