# Supplementary material for: Strengthening Jordan’s Laboratory Capacity for Communicable Diseases: A Comprehensive Multi-Method Mapping Toward Harmonized National Laboratories and Evidence-Informed Public Health Planning
Source: Int J Environ Res Public Health. 2025 Sep 20;22(9):1459. doi: 10.3390/ijerph22091459 (PMC12469349; doi:10.3390/ijerph22091459)
Supplement: Supplementary file 1 [file ijerph-22-01459-s001.zip › Supplementary File S1. List of Key Stakeholders.pdf]

Supplementary File S1. List of Key Stakeholders

Stakeholder Name	Department Name
**Ministry of Health (MoH)**	Communicable Diseases Directorate
	Chest Diseases, Foreigners Health and Occupational Health Directorate
	Directorate of Blood Bank
	Directorate of Laboratories
	Al Bashir Hospital
	Al Karak Hospital
	Malaria Laboratory
**Royal Medical Services (RMS)**	Princess Eman Research Center
	Princess Alia Hospital
	Prince Rashid Hospital
**Jordan Armed Forces (JAF)**	Central Quality Laboratories
**The University of Jordan (JU) and The University of Jordan Hospital (JUH)**	Toxicology Laboratory/JU
	Water Safety Laboratory/JU
	Infection Control Department/ JUH
	Department of Immunology and Microbiology /JUH
**King Abdullah University Hospital (KAUH)**	Infection Control Department
	Laboratory Department
**King Hussein Cancer Center (KHCC)**	Infection Control Department
	Laboratory Department
**Private Sector**	Speciality Hospital
	Biolab
	Ibn Hayan Lab
	Jordanian Medical Laboratory Specialists Association
	Jordanian Society for Laboratory Medical Sciences
	Medical Laboratories and Analysis Association
**Jordan University of Science and Technology (JUST)**	Infection Control Department
	Department of Pathology and Microbiology<br>Veterinary Medicine
	Princess Haya Center for Biotechnology
**Jordan Food and Drug Administration (JFDA)**	Food & Drug Laboratory

**Jordan Standards and Metrology Organization (JSMO)**	Central Laboratory
**Ministry of Agriculture (MoA)**	Veterinary and Animal Health Directorate
	Central Veterinary Laboratory
**Ministry of Environment (MoEnv)**	Environment Surveillance and Assessment Directorate
**Water Authority of Jordan (WAJ)**	Water Quality Laboratories
**Royal Scientific Society (RSS)**	Microbiology Laboratory
**World Health Organization (WHO)**	Infectious Diseases Expert
	Laboratory Capacity Building Unit
**Eastern Mediterranean Public Health Network (EMPHNET)**	Disease Control and Prevention Unit
**Civilian Research and Development Foundation (CRDF)**	Laboratory Program
**The United Nations Relief and Works Agency for Palestine Refugees in the Near East (UNRWA)**	Laboratories
