# Supplementary material for: Strengthening Jordan’s Laboratory Capacity for Communicable Diseases: A Comprehensive Multi-Method Mapping Toward Harmonized National Laboratories and Evidence-Informed Public Health Planning
Source: Int J Environ Res Public Health. 2025 Sep 20;22(9):1459. doi: 10.3390/ijerph22091459 (PMC12469349; doi:10.3390/ijerph22091459)
Supplement: Supplementary file 1 [file ijerph-22-01459-s001.zip › Supplementary File S2 - Semi Structured Interview Guide.pdf]

Supplementary File S2. Semi-Structed Interview Guide

General Information

- **Interviewee Name:**
- **Organization/Affiliation:**
- **Designation/Role:**
- **Date:**

Introduction

Thank you for participating in this key informant interview. The purpose of this survey is to gather information about the existing laboratory legislations, policies, and standard operating procedures (SOPs) for communicable diseases in Jordan. Your valuable insights will contribute to the mapping and review of the current landscape. Please note that all information provided will be treated confidentially.

Overview of Laboratory Legislations:

1. Are there specific legislations or regulations governing laboratories involved in the diagnosis and surveillance of communicable diseases in Jordan?
2. Can you provide an overview of the key legislative documents related to laboratory practices in this context?
3. Are there any recent updates or amendments to these legislations?

Laboratory Policies:

4. Are there specific policies or guidelines related to laboratory practices for communicable diseases in Jordan?
5. Can you describe the main policies that laboratories are expected to adhere to?
6. How are these policies disseminated and implemented within laboratory under your supervision?

Standard Operating Procedures & Communicable Diseases Testing Algorithms:

7. Are there standardized operating procedures in place for various laboratory processes involved in diagnosing and monitoring communicable diseases?
8. Are there standardized communicable disease testing algorithms standardized and available for laboratories?
9. Can you provide examples of key SOPs and testing algorithms that laboratories commonly follow?
10. How are these SOPs and testing algorithms developed, updated, Approved, disseminated, reviewed, and communicated to laboratories?
11. Who is responsible for developing algorithms?
12. Are there standardized operating procedures in place for biosafety and biosecurity measures within the laboratory? Who monitors compliance?
13. Are there standardized operating procedures in place for quality control within the laboratory? Who monitors compliance?

Compliance and Enforcement:

14. How is compliance with laboratory legislations, policies, SOPs and testing algorithms monitored?
15. Are there any regulatory or oversight bodies responsible for enforcing these standards?
16. What are the consequences for non-compliance?

Challenges and Gaps:

17. In your opinion, what are the key challenges or gaps in the existing laboratory legislations or policies for communicable diseases in Jordan?
18. Are there any areas where further improvements or enhancements are needed?
19. How can the current system be strengthened to ensure effective management of communicable diseases?

Collaboration and Coordination:

20. Are there any collaborative efforts or partnerships between different stakeholders involved in laboratory practices for communicable diseases?
21. How is coordination maintained among relevant organizations and agencies to ensure a standardized approach?
22. Are there any forums or platforms where discussions and knowledge sharing on laboratory practices take place?
23. Are there any joint training programs?

Future Direction:

24. Based on your expertise, what are the key areas or aspects that should be prioritized in the future to strengthen laboratory legislations, policies, and SOPs for communicable diseases in Jordan?
25. Are there any emerging trends or technological advancements that should be considered?

Closing:

26. Is there any additional information or insights you would like to share regarding laboratory legislations, policies, testing algorithms or SOPs for communicable diseases in Jordan?

Thank you for your time and valuable input. Your contributions are greatly appreciated.
