# Supplementary material for: Strengthening Jordan’s Laboratory Capacity for Communicable Diseases: A Comprehensive Multi-Method Mapping Toward Harmonized National Laboratories and Evidence-Informed Public Health Planning
Source: Int J Environ Res Public Health. 2025 Sep 20;22(9):1459. doi: 10.3390/ijerph22091459 (PMC12469349; doi:10.3390/ijerph22091459)
Supplement: Supplementary file 1 [file ijerph-22-01459-s001.zip › Supplementary File S3. Field Visit Tool.pdf]

Supplementary File S3. Field Visit Tool

General Information	
**Organization Name**	
**Governorate Name**	
**Visit Date/ phone call interview**	
**Name of Assessor**	
**Name and title of Focal Person**	
**Type of Facility**	▪ Governmental<br>▪ Military<br>▪ University<br>▪ Private<br>▪ NGO
**Type of Laboratory Service**	▪ Clinical Laboratory<br>▪ Blood Banking Laboratory<br>▪ Disease Surveillance and Control Laboratory<br>▪ Animal Health Laboratory<br>▪ Environmental Health Laboratory<br>▪ Food Safety Laboratory

Governance & Leadership			
**Available**		**Not Available**	**If Yes, Specify**
**Organizational Structure**	☐	☐	
**Strategic Plan**	☐	☐	
**Key Performance Indicators (KPIs)**	☐	☐	
**Accreditation Program**	☐	☐	

Technical Operations	
**1. National Guidelines and Protocols**	
Are national guidelines and protocols for laboratory testing and result reporting of communicable disease available in the lab?	▪ Yes<br>▪ No<br>▪ Not sure, don't know
Is the lab adhering to these guidelines?	▪ Yes<br>▪ No<br>▪ Not sure, don't know

Are they accessible to relevant staff?	▪ Yes<br>▪ No<br>▪ Not sure, don't know
Does related staff acknowledge guidelines?	▪ Yes<br>▪ No<br>▪ Not sure, don't know
Did relevant staff receive training on related practices?	▪ Yes<br>▪ No<br>▪ Not sure, don't know
Are all records of training available?	▪ Yes<br>▪ No<br>▪ Not sure, don't know

2. Communicable Disease Testing

Is there ongoing/published research conducted at the lab?	▪ Yes<br>▪ No<br>▪ Not sure, don't know<br>▪ -IF yes, specify…...
What's the electronic systems applied within the lab?	
Which of the following communicable disease testing is performed at the lab?	

#	Category	Communicable disease	Test Performed?	If performed, SOP Available?	Testing Methodology/ Technology used
1	Respiratory Tract Infections, meningitis, ear infections and systemic infections	▪ Bacterial Identification and Sensitivity Testing	▪ Yes<br>▪ No	▪ Yes<br>▪ No	▪
		▪ COVID-19	▪ Yes<br>▪ No	▪ Yes<br>▪ No	▪
		▪ CMV	▪ Yes<br>▪ No	▪ Yes<br>▪ No	▪
		▪ Enterovirus	▪ Yes<br>▪ No	▪ Yes<br>▪ No	▪
		▪ EBV	▪ Yes<br>▪ No	▪ Yes<br>▪ No	▪
		▪ Influenza	▪ Yes<br>▪ No	▪ Yes<br>▪ No	▪
		▪ Tuberculosis	▪ Yes<br>▪ No	▪ Yes<br>▪ No	▪
		▪ Legionella	▪ Yes	▪ Yes	▪

			▪ No	▪ No	
		▪ Meningococci	▪ Yes<br>▪ No	▪ Yes<br>▪ No	▪
		▪ Hemophilus	▪ Yes<br>▪ No	▪ Yes<br>▪ No	▪
		▪ HACEK group	▪ Yes<br>▪ No	▪ Yes<br>▪ No	▪
		▪ Periodontal streptococci	▪ Yes<br>▪ No	▪ Yes<br>▪ No	▪
		▪ Urinary Tract Infection	▪ Yes<br>▪ No	▪ Yes<br>▪ No	▪
2	Sexually Transmitted & Blood borne Diseases	▪ HIV	▪ Yes<br>▪ No	▪ Yes<br>▪ No	▪
		▪ Syphilis	▪ Yes<br>▪ No	▪ Yes<br>▪ No	▪
		▪ Chlamydia.	▪ Yes<br>▪ No	▪ Yes<br>▪ No	▪
		▪ Gonorrhea.	▪ Yes<br>▪ No	▪ Yes<br>▪ No	▪
		▪ Human papilloma virus HPV	▪ Yes<br>▪ No	▪ Yes<br>▪ No	▪
		▪ Hepatitis B, C (HCV, HBV)	▪ Yes<br>▪ No	▪ Yes<br>▪ No	▪
		▪ Mycoplasma	▪ Yes<br>▪ No	▪ Yes<br>▪ No	▪
		▪ Trichomonas Vaginalis	▪ Yes<br>▪ No	▪ Yes<br>▪ No	▪
3	Gastrointestinal Infections and food poisoning	▪ Salmonellosis	▪ Yes<br>▪ No	▪ Yes<br>▪ No	▪
		▪ Shigellosis	▪ Yes<br>▪ No	▪ Yes<br>▪ No	▪
		▪ Enterohemorrhagic E. coli and other strains	▪ Yes<br>▪ No	▪ Yes<br>▪ No	▪
		▪ Campylobacteriosis	▪ Yes<br>▪ No	▪ Yes<br>▪ No	▪
		▪ Food intoxication (Bacillus, Staphylococci)	▪ Yes<br>▪ No	▪ Yes<br>▪ No	▪
		▪ Hepatitis A viruses	▪ Yes<br>▪ No	▪ Yes<br>▪ No	▪
		▪ Cholera	▪ Yes<br>▪ No	▪ Yes<br>▪ No	▪
		▪ Rota virus	▪ Yes<br>▪ No	▪ Yes<br>▪ No	▪<br>▪
		▪ Norwalk virus	▪ Yes<br>▪ No	▪ Yes<br>▪ No	▪

		▪ Helicobacter	▪ Yes<br>▪ No	▪ Yes<br>▪ No	▪
4	Skin and rash lesions	▪ Bacteria identification	▪ Yes<br>▪ No	▪ Yes<br>▪ No	▪
		▪ Rubella	▪ Yes<br>▪ No	▪ Yes<br>▪ No	▪
		▪ Parvovirus	▪ Yes<br>▪ No	▪ Yes<br>▪ No	▪
		▪ Roseola	▪ Yes<br>▪ No	▪ Yes<br>▪ No	▪
		▪ Herpes	▪ Yes<br>▪ No	▪ Yes<br>▪ No	▪
		▪ Measles	▪ Yes<br>▪ No	▪ Yes<br>▪ No	▪
		▪ Chicken pox	▪ Yes<br>▪ No	▪ Yes<br>▪ No	▪
5	Neurotoxicity	▪ Tetanus	▪ Yes<br>▪ No	▪ Yes<br>▪ No	▪
		▪ Botulism	▪ Yes<br>▪ No	▪ Yes<br>▪ No	▪
		▪ Rabies	▪ Yes<br>▪ No	▪ Yes<br>▪ No	▪
		▪ Bovine Spongiform Encephalopathy (BSE)	▪ Yes<br>▪ No	▪ Yes<br>▪ No	▪
		▪ Polio	▪ Yes<br>▪ No	▪ Yes<br>▪ No	▪
		▪ Guillain bare	▪ Yes<br>▪ No	▪ Yes<br>▪ No	▪
6	Fever and systemic and lymphadenopathic infections	▪ Brucellosis	▪ Yes<br>▪ No	▪ Yes<br>▪ No	▪
		▪ Typhoid fever	▪ Yes<br>▪ No	▪ Yes<br>▪ No	▪
		▪ Relapsing fever	▪ Yes<br>▪ No	▪ Yes<br>▪ No	▪
		▪ Infectious mononucleosis	▪ Yes<br>▪ No	▪ Yes<br>▪ No	▪
		▪ Leptospirosis and Lyme disease	▪ Yes<br>▪ No	▪ Yes<br>▪ No	▪
		▪ Rose spot fever and rickettsia diseases	▪ Yes<br>▪ No	▪ Yes<br>▪ No	▪
		▪ Hemorrhagic viral fever and Ebola, west Nile, Norovirus	▪ Yes<br>▪ No	▪ Yes<br>▪ No	▪
		▪ Q Fever	▪ Yes<br>▪ No	▪ Yes<br>▪ No	▪

7	Mycosis	▪ Dermatophytes, Tinea and cutaneous mycosis	▪ Yes<br>▪ No	▪ Yes<br>▪ No	▪
		▪ Candidiasis	▪ Yes<br>▪ No	▪ Yes<br>▪ No	▪
		▪ Systemic mycosis (Cryptococcus)	▪ Yes<br>▪ No	▪ Yes<br>▪ No	▪
8	Parasites – Intestinal	▪ Amebiasis	▪ Yes<br>▪ No	▪ Yes<br>▪ No	▪
		▪ Giardiasis	▪ Yes<br>▪ No	▪ Yes<br>▪ No	▪
		▪ Cryptosporidiosis	▪ Yes<br>▪ No	▪ Yes<br>▪ No	▪
		▪ Pinworm	▪ Yes<br>▪ No	▪ Yes<br>▪ No	▪
		▪ Ascaris	▪ Yes<br>▪ No	▪ Yes<br>▪ No	▪
		▪ Tape worms	▪ Yes<br>▪ No	▪ Yes<br>▪ No	▪
9	Parasites – blood, lymph and others	▪ Malaria	▪ Yes<br>▪ No	▪ Yes<br>▪ No	▪
		▪ Toxoplasmosis	▪ Yes<br>▪ No	▪ Yes<br>▪ No	▪
		▪ Hydatid Disease (Cyst)	▪ Yes<br>▪ No	▪ Yes<br>▪ No	▪
		▪ Chagas disease	▪ Yes<br>▪ No	▪ Yes<br>▪ No	▪
		▪ Leishmaniasis	▪ Yes<br>▪ No	▪ Yes<br>▪ No	▪
		▪ Schistosoma	▪ Yes<br>▪ No	▪ Yes<br>▪ No	▪
		▪ Filaria	▪ Yes<br>▪ No	▪ Yes<br>▪ No	▪
		▪ Scabies	▪ Yes<br>▪ No	▪ Yes<br>▪ No	▪
		▪ Head lice and nets	▪ Yes<br>▪ No	▪ Yes<br>▪ No	▪

List of equipment for communicable disease testing at the lab:	Available?	If yes, Specify Count
▪ DNA Sequencer	▪ Yes<br>▪ No	
▪ PCR Machine	▪ Yes<br>▪ No	

▪ Automated Culture identification and antimicrobial susceptibility System	▪ Yes<br>▪ No	
▪ ELISA Plate reader	▪ Yes<br>▪ No	
▪ Fluorescent Microscope	▪ Yes<br>▪ No	
▪ Extraction Machines	▪ Yes<br>▪ No	
▪ Point of Care “gene expert, film array”	▪ Yes<br>▪ No	
▪ NGS (Next Generation Sequencer)	▪ Yes<br>▪ No	
▪ Blotting systems	▪ Yes<br>▪ No	
**3. Laboratory workflow**		
**4. Specimen receiving and handling**		
Are the following policies available in the lab?		
a. Specimen request	▪ Yes<br>▪ No<br>▪ Not sure, don’t know	
b. Specimen collection and handling	▪ Yes<br>▪ No<br>▪ Not sure, don’t know	
c. Specimen labeling	▪ Yes<br>▪ No<br>▪ Not sure, don’t know	
d. Specimen rejection	▪ Yes<br>▪ No<br>▪ Not sure, don’t know	
e. Specimen tracking	▪ Yes<br>▪ No<br>▪ Not sure, don’t know	

f. Specimen storage, retention and disposal	▪ Yes<br>▪ No<br>▪ Not sure, don't know
g. Reporting positive results	▪ Yes<br>▪ No<br>▪ Not sure, don't know
Are the policies accessible to staff?	▪ Yes<br>▪ No<br>▪ Not sure, don't know
Does related staff acknowledge policies?	▪ Yes<br>▪ No<br>▪ Not sure, don't know
Did relevant staff receive training on related practices?	▪ Yes<br>▪ No<br>▪ Not sure, don't know
Are all records of training available?	▪ Yes<br>▪ No<br>▪ Not sure, don't know
To whom do you report the positive results? Notification procedure?	
**Reagents**	
Are reagents, calibrators, controls, stains, chemicals, and solutions properly labeled, as applicable and appropriate, with the following elements:	
a. Content and quantity, concentration or titer	▪ Yes<br>▪ No<br>▪ Not sure, don't know
b. Storage requirements	▪ Yes<br>▪ No<br>▪ Not sure, don't know
c. Date prepared, filtered or reconstituted by laboratory	▪ Yes<br>▪ No<br>▪ Not sure, don't know
d. Expiration date	▪ Yes<br>▪ No<br>▪ Not sure, don't know
Are reagents and kits used, handled, and stored per manufacturer instruction?	▪ Yes<br>▪ No<br>▪ Not sure, don't know
Are temperature of storage areas being monitored	▪ Yes<br>▪ No<br>▪ Not sure, don't know

Are all reagents (e.g., chemicals, stains, media, antibodies) are used within their indicated expiration date?	▪ Yes<br>▪ No<br>▪ Not sure, don't know
Is there a system to track and monitor inventory of kits, reagents, media, etc. to ensure availability and prevent stockouts?	▪ Yes<br>▪ No<br>▪ Not sure, don't know
**4. Standard Operating Procedures (SOPs)**	
Are there documented SOPs for infectious disease tests performed at the lab?	▪ Yes<br>▪ No<br>▪ Not sure, don't know
Does the SOP provide a list of essential supplies (reagents and consumables)?	▪ Yes<br>▪ No<br>▪ Not sure, don't know
Does the SOP provide a list of essential equipment?	▪ Yes<br>▪ No<br>▪ Not sure, don't know
Are SOPs accessible to all relevant staff?	▪ Yes<br>▪ No<br>▪ Not sure, don't know
Does related staff acknowledge SOPs?	▪ Yes<br>▪ No<br>▪ Not sure, don't know
Are SOPs reviewed, approved/ signed by authorizing entity?	▪ Yes<br>▪ No<br>▪ Not sure, don't know
Who is responsible for disseminating policies, SOPs, and protocols to the clinical wards within hospitals?	
**5. Quality Control Measures**	
Are there written quality assurance policies and procedures available in the laboratory?	▪ Yes<br>▪ No<br>▪ Not sure, don't know
Are they accessible to relevant staff?	▪ Yes<br>▪ No<br>▪ Not sure, don't know
Does related staff acknowledge them?	▪ Yes<br>▪ No<br>▪ Not sure
Is there a quality control officer?	▪ Yes<br>▪ No

Does the laboratory undertake the following internal quality control procedures?	
a. Check each batch of reagents using known positive and negative specimens?	▪ Yes<br>▪ No<br>▪ Not sure, don't know
b. Include commercially prepared controls whenever a batch of tests is run?	▪ Yes<br>▪ No<br>▪ Not sure, don't know
Does the laboratory participate in any external quality assurance scheme (Proficiency Testing)?	▪ Yes<br>▪ No<br>▪ Not sure, don't know
If yes, which scheme? How often a year?	
Are corrective actions taken based on quality control data?	▪ Yes<br>▪ No<br>▪ Not sure, don't know
**Method validation/ verification**	
Are there policies for method validation/ verification available in the lab?	▪ Yes<br>▪ No<br>▪ Not sure, don't know
Are they accessible to relevant staff?	▪ Yes<br>▪ No<br>▪ Not sure, don't know
Does related staff acknowledge policies?	▪ Yes<br>▪ No<br>▪ Not sure, don't know
Are manufacturer instructions followed as per kit leaflet?	▪ Yes<br>▪ No<br>▪ Not sure, don't know
▪ Are test methods validated/ verified for:	
Analytical accuracy	▪ Yes<br>▪ No<br>▪ Not sure, don't know
Analytical precision	▪ Yes<br>▪ No<br>▪ Not sure, don't know
Are method validation/verification studies approved and signed by authorizing entities?	▪ Yes<br>▪ No<br>▪ Not sure, don't know
Did staff receive training on method validation/ verification?	▪ Yes<br>▪ No<br>▪ Not sure, don't know
Are all records of training available?	▪ Yes<br>▪ No

	▪ Not sure, don't know
**6. Communicable Diseases Testing Algorithms**	
Are communicable disease testing algorithms available in the laboratory, hospital clinical wards, or electronic systems?	▪ Yes<br>▪ No<br>▪ Not sure, don't know
Are they accessible to all relevant staff?	▪ Yes<br>▪ No<br>▪ Not sure, don't know
Does related staff acknowledge the testing algorithms?	▪ Yes<br>▪ No<br>▪ Not sure, don't know
Did staff receive training on the proper usage of testing algorithms?	▪ Yes<br>▪ No<br>▪ Not sure, don't know
Are all records of training available?	▪ Yes<br>▪ No<br>▪ Not sure, don't know
**7. Safety and Security Measures**	
Are there written guidelines on safety precautions?	
a. Infection prevention	▪ Yes<br>▪ No<br>▪ Not sure, don't know
b. Safe disposal of sharps	▪ Yes<br>▪ No<br>▪ Not sure, don't know
c. Use of PPEs	▪ Yes<br>▪ No<br>▪ Not sure, don't know
d. Safe disposal of biohazardous medical waste	▪ Yes<br>▪ No<br>▪ Not sure, don't know
Is there an assigned safety Officer within the laboratory?	▪ Yes<br>▪ No
Are laboratory personnel provided with and wearing appropriate PPE?	▪ Yes<br>▪ No<br>▪ Not sure, don't know
Is there a process to train and educate laboratory personnel on the proper use, maintenance, and disposal of PPE?	▪ Yes<br>▪ No<br>▪ Not sure, don't know

Are there clear and visible signs indicating biohazardous areas and appropriate safety precautions?	▪ Yes<br>▪ No<br>▪ Not sure, don't know
Are Safety Data Sheets (SDS) available for hazardous materials used in the laboratory?	▪ Yes<br>▪ No<br>▪ Not sure, don't know
Is staff aware of their hazard and acknowledges SDSs?	▪ Yes<br>▪ No<br>▪ Not sure, don't know
Are biological safety cabinets (BSCs) available and properly maintained in the laboratory?	▪ Yes<br>▪ No<br>▪ Not sure, don't know
Is there an Autoclave available within the Laboratory?	▪ Yes<br>▪ No<br>▪ Not sure, don't know
Are BSCs certified and inspected regularly according to regulatory requirements?	▪ Yes<br>▪ No<br>▪ Not sure, don't know
Is there a documented waste management plan in place for the proper segregation, storage, and disposal of different types of waste? - Check autoclave/incinerator-	▪ Yes<br>▪ No<br>▪ Not sure, don't know
Are biohazardous waste, sharps, and chemical waste containers properly labeled and located in designated areas?	▪ Yes<br>▪ No<br>▪ Not sure, don't know
Is there a process to ensure the safe transport and disposal of hazardous waste in compliance with local regulations?	▪ Yes<br>▪ No<br>▪ Not sure, don't know
Is there a comprehensive safety training program in place for laboratory personnel?	▪ Yes<br>▪ No<br>▪ Not sure, don't know
Are all records of training available?	▪ Yes<br>▪ No<br>▪ Not sure, don't know
Is there a policy for staff exposure follow up?	▪ Yes<br>▪ No<br>▪ Not sure, don't know
**For Microbiology Laboratory**	
**Culture Media Preparation**	

Is there a designated area for media preparation that is clean and free from potential contaminants?	▪ Yes<br>▪ No<br>▪ Not sure, don’t know
Are all media components and reagents properly labeled and within their expiration dates?	▪ Yes<br>▪ No<br>▪ Not sure, don’t know
Are media components and reagents stored according to the manufacturer's instructions?	▪ Yes<br>▪ No<br>▪ Not sure, don’t know
Is there a validated sterilization process for media preparation, such as autoclaving or filtration?	▪ Yes<br>▪ No<br>▪ Not sure, don’t know
Are sterilization parameters (e.g., temperature, pressure, time) monitored and documented?	▪ Yes<br>▪ No<br>▪ Not sure, don’t know
Are appropriate quality control measures performed on media batches, including growth promotion testing and sterility testing?	▪ Yes<br>▪ No<br>▪ Not sure, don’t know
Are there written SOPs for media preparation that are followed consistently?	▪ Yes<br>▪ No<br>▪ Not sure, don’t know
Is aseptic technique strictly followed during media preparation to minimize the risk of contamination?	▪ Yes<br>▪ No<br>▪ Not sure, don’t know
Are environmental controls in place, such as laminar flow hoods or biological safety cabinets, to maintain a sterile working environment?	▪ Yes<br>▪ No<br>▪ Not sure, don’t know
**Culture and sensitivity testing**	
Are there written SOPs for culture and sensitivity testing that are followed consistently?	▪ Yes<br>▪ No<br>▪ Not sure, don’t know
Is there a systematic process for isolating and identifying microorganisms from clinical specimens?	▪ Yes<br>▪ No<br>▪ Not sure, don’t know
Is there a mechanism to ensure accurate identification of isolates using reliable and validated methods?	▪ Yes<br>▪ No<br>▪ Not sure, don’t know
Are appropriate methods and interpretive criteria used for performing Antimicrobial Susceptibility Testing (AST)/Antibiogram, such as disk diffusion, broth microdilution, or automated systems?	▪ Yes<br>▪ No<br>▪ Not sure, don’t know

Are AST results interpreted and reported according to established guidelines, such as CLSI or EUCAST?	▪ Yes<br>▪ No<br>▪ Not sure, don't know
Are quality control strains and panels used to validate and monitor the performance of AST methods?	▪ Yes<br>▪ No<br>▪ Not sure, don't know
Are results communicated in a clear, concise, and timely manner to healthcare providers?	▪ Yes<br>▪ No<br>▪ Not sure, don't know
Is there a training program in place for new employees and ongoing training for existing staff on Microbiology, serology, and molecular testing?	▪ Yes<br>▪ No<br>▪ Not sure, don't know
