# Supplementary material for: Strengthening Jordan’s Laboratory Capacity for Communicable Diseases: A Comprehensive Multi-Method Mapping Toward Harmonized National Laboratories and Evidence-Informed Public Health Planning
Source: Int J Environ Res Public Health. 2025 Sep 20;22(9):1459. doi: 10.3390/ijerph22091459 (PMC12469349; doi:10.3390/ijerph22091459)
Supplement: Supplementary file 1 [file ijerph-22-01459-s001.zip › Supplementary File S4. General Information on Participating Labs.pdf]

Supplementary File S4. General Information on Participating Labs

**Lab Category**	**Number of Labs**	**Governorate**				**Sector**				
		**Amman**	**Irbid**	**Karak**	**Aqaba**	**Govern-mental**	**Private**	**University**	**Military**	**NGO**
Clinical Laboratories	15	11	3	1		5	2	3	4	1
Animal Health Laboratories	1	1				1				
Environmental Health and/ or food safety Labs	6	5			1	4		1		1
Blood Banking Laboratories	1	1				1				
**Total**	**23**									
