# Supplementary material for: Strengthening Jordan’s Laboratory Capacity for Communicable Diseases: A Comprehensive Multi-Method Mapping Toward Harmonized National Laboratories and Evidence-Informed Public Health Planning
Source: Int J Environ Res Public Health. 2025 Sep 20;22(9):1459. doi: 10.3390/ijerph22091459 (PMC12469349; doi:10.3390/ijerph22091459)
Supplement: Supplementary file 1 [file ijerph-22-01459-s001.zip › Supplementary File S5. Conducted Field Visits.pdf]

**Supplementary File S5**. Conducted Field Visits

| No | Entity | Date of visit | Governorate | Type of facility | Type of lab service |
|---|---|---|---|---|---|
| 1 | Water, Energy and Environment Center (WEEC) at the University of Jordan | 8/12/2023 | Amman | University | Environmental water testing |
| 2 | MoA Animal Wealth Lab | 8/13/2023 | Amman | Governmental | Animal health |
| 3 | Water Authority of Jordan (WAJ) | 8/14/2023 | Amman | Governmental | Environmental water testing |
| 4 | Biolab | 8/20/2023 | Amman | Private | Human health |
| 5 | Jordan Food and Drug Administration (JFDA) | 8/21/2023 | Amman | Governmental | Food safety |
| 6 | King Abdullah University Hospital (KAUH) | 8/23/2023 | Irbid | University | Human health |
| 7 | Princess Haya Biotechnology Centre (PHBC) | 8/23/2023 | Irbid | University | Human Health & Research |
| 8 | Jordan University Hospital | 8/24/2023 | Amman | University | Human heath |
| 9 | MoH Environmental Health Directorate | 8/27/2023 | Amman | Governmental | Environmental water testing |
| 10 | MoH Tuberculosis (TB) Lab | 8/28/2023 | Amman | Governmental | Human health |
| 11 | Royal Science Society (RSS) | 8/29/2023 | Amman | NGO | Environmental water testing & food safety |

| No | Entity | Date of visit | Governorate | Type of facility | Type of lab service |
|---|---|---|---|---|---|
| 12 | Ben Hayan Lab | 8/31/2023 | Aqaba | Governmental | Environmental water testing & food safety |
| 13 | Al Bashir Hospitals | 9/3/2023 | Amman | Governmental | Human health |
| 14 | National Blood Bank Directorate | 9/3/2023 | Amman | Governmental | Blood banking |
| 15 | MoH Central Lab | 9/4/2023 | Amman | Governmental | Human health |
| 16 | Al Karak Hospital | 9/5/2023 | Karak | Governmental | Human health |
| 17 | King Hussein Cancer Centre (KHCC) | 9/6/2023 | Amman | NGO | Human health |
| 18 | Royal Hospital | 9/7/2023 | Amman | Private | Human health |
| 19 | MoH Malaria and Bilharzia Lab | 9/10/2023 | Amman | Governmental | Human health |
| 20 | Queen Alia Hospital | 9/18/2023 | Amman | Military | Human health |
| 21 | Princess Iman Research and laboratory Sciences Center | 9/19/2023 | Amman | Military | Human health |
| 22 | Military Mobile Laboratory | 9/19/2023 | Amman | Military | Human health |
| 23 | Prince Rashed Hospital | 9/20/2023 | Irbid | Military | Human health |
